# Genetic Parameters of Bovine Milk Fatty Acid Profile, Yield, Composition, Total and Differential Somatic Cell Count

**DOI:** 10.3390/ani10122406

**Published:** 2020-12-16

**Authors:** Tania Bobbo, Mauro Penasa, Martino Cassandro

**Affiliations:** Department of Agronomy, Food, Natural resources, Animals and Environment (DAFNAE), University of Padova, Viale dell’Università 16, 35020 Legnaro, Italy; mauro.penasa@unipd.it (M.P.); martino.cassandro@unipd.it (M.C.)

**Keywords:** milk fatty acid, heritability, selection, somatic cell, dairy cattle

## Abstract

**Simple Summary:**

In the last years, consumers have shown a remarkable interest in the qualitative and healthy aspects of milk, leading to an increase of the demand for dairy products with high nutritional value. By applying a genetic approach, it is possible to improve the quality of the fat contained in milk and its derivatives. In the present study we explored the genetic aspects of bovine milk fatty acids, in order to evaluate the feasibility of including them in breeding programs to alter their proportions in milk and improve the nutritional and healthy quality of fat. Our results indicate that genetic selection on fatty acid profile is feasible, as fatty acids present sufficient genetic variability and are moderately heritable. However, in the light of estimated correlations with other milk traits, a clear breeding objective should first be established.

**Abstract:**

The growing interest of consumers for milk and dairy products of high nutritional value has pushed researchers to evaluate the feasibility of including fatty acids (FA) in selection programs to modify milk fat profile and improve its nutritional quality. Therefore, the aim of this study was to estimate genetic parameters of FA profile predicted by mid-infrared spectroscopy, milk yield, composition, and total and differential somatic cell count. Edited data included 35,331 test-day records of 25,407 Italian Holstein cows from 652 herds. Variance components and heritability were estimated using single-trait repeatability animal models, whereas bivariate repeatability animal models were used to estimate genetic and phenotypic correlations between traits, including the fixed effects of stage of lactation, parity, and herd-test-date, and the random effects of additive genetic animal, cow permanent environment and the residual. Heritabilities and genetic correlations obtained in the present study reflected both the origins of FA (extracted from the blood or synthesized de novo by the mammary gland) and their grouping according to saturation or chain length. In addition, correlations among FA groups were in line with correlation among individual FA. Moderate negative genetic correlations between FA and milk yield and moderate to strong positive correlations with fat, protein, and casein percentages suggest that actual selection programs are currently affecting all FA groups, not only the desired ones (e.g., polyunsaturated FA). The absence of association with differential somatic cell count and the weak association with somatic cell score indicate that selection on FA profile would not affect selection on resistance to mastitis and vice versa. In conclusion, our findings suggest that genetic selection on FA content is feasible, as FA are variable and moderately heritable. Nevertheless, in the light of correlations with other milk traits estimated in this study, a clear breeding goal should first be established.

## 1. Introduction

In the last years, the qualitative and healthy aspects of milk have aroused considerable interest in the consumers, with the increase in the demand for dairy products with high nutritional value. As reported by Haug et al. [1], the composition of the fat contained in milk, and in particular the content of oleic acid, conjugated linoleic acid, omega-3 fatty acids (FA), short- and medium-chain FA, can be associated with positive effects on health, pushing the consumers to pay particular attention to the quality of fat, especially in terms of FA profile. Indeed, the changes in eating habits over the last century have led to an increase in fat consumption and an imbalance in the intake ratios of the different FA, favoring Omega-6 FA (present in vegetable oils on an industrial level) and reducing Omega-3 FA, known for their anti-atherogenic, anti-thrombotic and anti-inflammatory properties [2].

Milk lipids are mainly composed of triglycerides, made up of one glycerol molecule and three FA molecules. The high number of FA esterified with glycerol identified in cow’s milk makes this food complex from the point of view of the lipid profile [3]. The fat component of milk, responsible for many organoleptic and technological properties of the milk itself and its derivatives, is influenced by various factors, including the breed of the animal, the diet, the type of farming system and the stage of lactation [4]. In addition to changes in the diet of animals, which allow to obtain short-term variations in the acidic profile of milk, it is possible to apply a genetic approach to improve the quality of the fat contained in milk and dairy products. This strategy, which allows for long-term improvements in the population, consists in genetically selecting the breeding animals in order to obtain milk with FA profile favorable to human health, e.g., rich in Omega-3 and polyunsaturated FA (PUFA) and with low concentrations of saturated FA (SFA). In fact, while an excess of SFA can increase the risk of cardiovascular disease, PUFA are considered "good fats" as they favor the replacement of LDL with HDL cholesterol [1].

Several studies have reported the presence of genetic variability of the FA profile of milk and consequently the possibility of improving the profile itself through genetic selection [5,6,7]. Nevertheless, correlations with other milk traits should be taken into account to use FA profile for genetic purposes. For this reason, several studies have explored the phenotypic and genetic relationships of milk FA with yield and composition [6,8,9]. In addition, the genetic aspects of FA and their possible use in genomic selection have been investigated also in sheep [10,11] and goat [12,13] milk. Recently, the laboratory of the Breeders Association of Veneto Region (Padova, Italy) has expanded the range of information provided in the frame of the monthly milk recording procedures, including the FA profile in addition to more traditional milk composition traits such as fat, protein, casein, lactose and urea content. Moreover, the recent implementation of new advanced milk-testing technology has allowed measurement of both traditional somatic cell count (SCC) and new differential somatic cell count (DSCC), which is the ratio of neutrophils plus lymphocytes to total SCC [14]. This novel udder health trait provides more detailed insights on the inflammatory status of the mammary gland and the combined use of SCC and DSCC has been proposed as a novel approach to screen for udder health status [15].

Therefore, the aim of the present study was to explore the genetic correlations among FA, and between FA profile and several milk traits. To the best of our knowledge, this is the first study exploring the relationships between FA and the new DSCC.

## 2. Materials and Methods

### 2.1. Data and Editing

Test-day milk records of Italian Holstein cows collected from July 2019 to January 2020 within the routine milk recording system were provided by the Breeders Association of Veneto Region (Padova, Italy). Data included milk yield (kg/day); FA profile (i.e., SFA, monounsaturated FA (MUFA), PUFA, short-chain FA (SCFA), medium-chain FA (MCFA), long-chain FA (LCFA), *trans* FA (TFA), C14:0, C16:0, C18:0, and C18:1, measured as g/100 g milk), fat (%), protein (%), casein (%), lactose (%) and urea (mg/100 mL) measured using the MilkoScan FT6000 (Foss, Hillerød, Denmark); SCC (cells/mL) and DSCC (%) measured using the new Fossomatic 7 DC (Foss, Hillerød, Denmark). Somatic cell count was log-transformed to somatic cell score (SCS), according to Ali and Shook [16]. It is worth mentioning that, at the time of sampling, the laboratory of the Breeders Association of Veneto Region was equipped with one Fossomatic 7 DC, which provided information also on the new DSCC, and two Fossomatic 6 (Foss, Hillerød, Denmark). Indeed, approximately one third of the milk samples collected in the frame of the milk testing procedure of Veneto Region and processed by the laboratory could be randomly analysed for all traits investigated in the present study. Furthermore, the original dataset was edited to select cows from 5 to 480 days in milk and with known sire and dam. Contemporary groups, defined as cows sampled in the same herd and day (herd-test-date, HTD), with less than 3 cows were removed. For each trait, values exceeding three standard deviations from the respective mean were considered outliers and labelled as missing values in the dataset. After editing, 35,331 test-day records of 25,407 cows in 652 herds were available for subsequent analysis. All previous editing steps explain the low average number of TD per cow. Pedigree information was provided by the Italian Holstein and Jersey Association (Cremona, Italy), and included cows with phenotypic records and all their ancestors for a total of 115,581 animals.

### 2.2. Statistical Analysis

A preliminary analysis of variance was conducted to identify nongenetic factors affecting the investigated traits using the MIXED procedure of SAS software ver. 9.4 (SAS Institute Inc., Cary, NC, USA). The model included stage of lactation, parity, and HTD as fixed effects and cow as random effect, using the RANDOM statement of the PROC MIXED of SAS. Genetic analyses were then performed using the software VCE ver. 6.0 [17]. Variance components and heritability of the investigated traits were estimated using single-trait repeatability animal models. Heritability was computed as the ratio between additive genetic variance and total phenotypic variance. The latter was the sum of additive genetic, cow permanent environment and residual variances. Genetic and phenotypic correlations between the traits were estimated through bivariate repeatability animal models. In all cases, models used for genetic analyses included the fixed effects of stage of lactation (14 levels; classes of 30 days each, with the last being a class >395 days in milk), parity (4 levels: 1, 2, 3, and ≥4) and HTD (1160 levels), and the random effects of additive genetic animal, cow permanent environment and the residual.

## 3. Results and discussion

### 3.1. Descriptive Statistics, Variance Components, and Heritability

Saturated FA accounted for 2.62% of milk, whereas MUFA and PUFA represented 0.98% and 0.10%, respectively (Table 1). Similar results were reported by Penasa et al. [18], who found mean values of 2.58%, 0.91%, and 0.09% for SFA, MUFA and PUFA, respectively. Compared to our findings, higher mean values for SFA, MUFA and PUFA were reported by other authors [6,19], possibly due to higher mean value of milk fat than in our study. Short chain FA, MCFA, and LCFA constituted 0.41%, 1.74%, and 1.33% of milk, whereas TFA represented only 0.05% (Table 1). Fleming et al. [20] reported similar mean values for SCFA (0.41 g/dL) and LCFA (1.40 g/dL), and a higher value for MCFA (2.09 g/dL). Among individual FA, the most represented was C16:0 (1.16%), followed by C18:1 (0.88%), C14:0 (0.46%) and C18:0 (0.39%), which corresponded to 31.1%, 23.6%, 12.4%, and 10.5% of the total FA, respectively. A similar rank among individual FA was reported by Lopez-Villalobos et al. [21], who found C16:0, C18:1, C14:0, and C18:0 to be 27.62%, 21.40%, 11.54%, and 12.14% of the total FA, respectively; the other individual FA represented less than 5% of the total FA. Considering other milk traits, Holstein cows had an average milk yield of 32.4 kg/day and the composition of their milk was comparable with findings of other studies on the same breed [22,23]. Mean SCS was 3.17 and DSCC averaged 67%. The proportion of macrophages can be calculated as 100%—DSCC, so that in the present study macrophages were on average 33%.

Heritability of SFA (0.20 ± 0.02) was higher than heritability of MUFA and PUFA (0.07 ± 0.01) (Table 1). Estimates of heritability of FA groups reported in the literature [7,18] were similar to our findings. The same heritability for SFA observed in the present study was estimated by Bilal et al. [24]; nevertheless, those authors reported heritability of 0.21 and 0.15 for MUFA and PUFA, respectively. Greater heritability for SFA (0.36) and MUFA (0.15) were estimated by Soyeurt et al. [19], as well as by Bastin et al. [6] (0.426, 0.212, and 0.298 for SFA, MUFA and PUFA, respectively). Short chain FA and MCFA had greater coefficient of additive genetic variation (CV_a_ of 10.1% and 9.3%, respectively) and greater heritability (0.18 ± 0.02 and 0.23 ± 0.01, respectively) than LCFA (CV_a_ of 5.8% and heritability of 0.07 ± 0.01; Table 1). Our findings seem to suggest a stronger genetic control on SCFA and MCFA than on LCFA, which can be explained by the different origin of FA. Indeed, SCFA and MCFA (C4:0 to C14:0, and almost half of C16:0) are synthesized de novo in the udder, and the two main enzymes involved in the metabolic pathway (acetyl-coenzyme A carboxylase and FA synthetase) seem to be partially under genetic control [6]. Moderate heritability estimates of SCFA (0.438), MCFA (0.434) and LCFA (0.199) were reported by Bastin et al. [6]. Similarly, heritability of 0.420, 0.499, and 0.261 were obtained for SCFA, MCFA and LCFA by Fleming et al. [20]. In the present study, heritability of TFA was very low (0.02 ± 0.01) but CV_a_ was 8.2% (Table 1). Heritabilities of the individual FA were in line with estimates of the FA groups: The greatest heritability was estimated for C14:0 and C16:0 (0.21 ± 0.02), followed by C18:0 (0.08 ± 0.01) and C18:1 (0.07 ± 0.01) (Table 1). The CV_a_ of individual FA ranged from 5.9% (C18:1) to 9.3% (C16:0). A slightly different pattern was observed by Soyeurt et al. [19], who reported heritability greater than 0.30 for C14:0, C16:0, and C18:0, and 0.05 for C18:1. Bastin et al. [6] obtained heritability greater than 0.40 for C14:0 and C16:0, but lower for C18:0 (0.231) and C18:1 (0.179).

Differences among studies could be explained by the number of samples analyzed, the structure of data (e.g., breed, primiparae or pluriparae, daily or lactation data, repeated measures or not) as well as by the definition of FA traits (expressed as concentration in milk or in fat, or weight percentage) and the model used for genetic parameters estimation (e.g., sire or animal model, inclusion of fixed or random effects). For instance, FA expressed as concentration in milk are on average more heritable than FA expressed in milk fat [6], due to the high heritability of fat content [5]. In addition, analytical methodologies for measuring FA (gas chromatography or mid-infrared spectrometry) could affect heritability estimates [25]. Indeed, it is difficult to compare heritability estimates among studies. Nevertheless, the presence of genetic variability and the low to moderate heritability estimates observed in the present study suggested that FA can be exploited for breeding purposes.

Heritability of milk production and composition traits ranged from 0.06 ± 0.01 (milk yield) to 0.28 ± 0.02 (casein) (Table 1). These findings are mostly in agreement with estimates reported by Bobbo et al. [22] for the same breed. Similar to previous studies [6,20], heritability of fat and protein contents was higher than that for milk yield. In accordance with the preliminary results of Bobbo et al. [22], heritability of DSCC was almost double the value of SCS (0.09 ± 0.01 and 0.05 ± 0.01, respectively), thus confirming the possible use of DSCC in genetic selection. To the best of our knowledge, this is the first study that has explored the genetic aspects of DSCC.

### 3.2. Genetic and Phenotypic Correlations among FA

Genetic and phenotypic correlations among groups of FA and individual FA are reported in Table 2. Saturated FA had stronger genetic correlation with MUFA (0.67 ± 0.03) rather than with PUFA (0.31 ± 0.07), whereas genetic correlation between MUFA and PUFA was 0.58 ± 0.06. Similar findings were reported by Soyeurt et al. [19] and Penasa et al. [18]. Bastin et al. [6] reported genetic correlations in the range from 0.60 (SFA and MUFA) to 0.73 (MUFA and PUFA). Nevertheless, those authors [6] reported approximate genetic correlations among traits as correlations among daily breeding values, possibly without fully representing the genetic relationships between traits. Although positive genetic correlations between SFA, MUFA, and PUFA have been reported in literature [19,26], some authors [9,27,28] reported strong negative genetic correlations. As explained by Lopez-Villalobos et al. [9], this discrepancy is due to the different expression of concentration of FA: as grams per 100 g of milk (positive correlation) or grams per 100 g of total FA (negative correlation). Considering the classification based on the length of the FA chain, medium to strong genetic correlations were estimated, with the strongest genetic and phenotypic associations between SCFA and MCFA (0.87 ± 0.01 and 0.81, respectively). A strong genetic correlation (>0.85) between SCFA and MCFA was observed also by Bastin et al. [6] and Narayana et al. [26], possibly due to their similar origin. Indeed, lower genetic correlations can be found between LCFA and both SCFA and MCFA, indicating different provenance of the FA groups. Lopez-Villalobos et al. [9], who expressed FA as % of total FA, observed a positive association between SCFA and MCFA (0.18) and negative association of LCFA with SCFA (−0.55) and MCFA (−0.09). Among individual FA, genetic correlations were in the range from 0.58 ± 0.05 (C16:0 and C18:1) to 0.79 ± 0.03 (C16:0 and C18:0), with the exception of a very strong genetic (and phenotypic) correlation of 0.94 ± 0.01 (0.92) between C14:0 and C16:0. Similar results were reported by Bastin et al. [6], with genetic correlations from 0.40 (C14:0 and C18:1) to 0.85 (C14:0 and C16:0). Genetic associations among C14:0, C16:0, and C18:0 could be explained by their similar origin, as these FA are synthesized de novo in the mammary gland [19]. Nevertheless, C16:0 and C18:0 are also partially extracted from the blood [19], explaining the moderate genetic correlations observed in this study among these FA and C18:1, which is also of blood origin. Krag et al. [27], which expressed concentrations as grams per 100 g of total FA, reported a weak positive genetic correlation between C14:0 and C16:0 (0.08) and negative correlations of C18:0 with both C14:0 (−0.37) and C16:0 (−0.18). Saturated FA had also strong genetic correlations with SCFA (0.93 ± 0.01) and MCFA (0.98 ± 0.00) and, among individual FA, with C14:0 (0.96 ± 0.01) and C16:0 (0.97 ± 0.00); phenotypic correlations were also greater than 0.90. These findings were expected, as almost 80% of SFA are composed by SCFA and MCFA [29]. Strong genetic correlations of SFA with SCFA (0.92), MCFA (0.99), C6:0 (0.91), C14:0 (0.93), C16:0 (0.97), and C17:0 (0.91) were estimated by Bastin et al. [6]. Strong genetic correlations (≥0.85) of SFA with C16:0 and C18:0 were reported also by Soyeurt et al. [19] and Petrini et al. [30]. Monounsaturated FA were strongly genetically and phenotypically associated with LCFA (0.91 ± 0.02 and 0.97, respectively), and, as expected, with C18:1 (0.98, both genetic and phenotypic correlation). Our results confirmed previous findings of Bastin et al. [6], who reported genetic correlations greater than 0.90 only with LCFA (0.93) and C18:1 (0.98), and of Petrini et al. [30], with a genetic correlation between MUFA and C18:1 of 0.95. Mele et al. [25], who expressed concentrations as grams per 100 g of total FA, reported strong negative genetic correlations of MUFA with C14:0 (−0.84 ± 0.10) and C16:0 (−0.89 ± 0.12), and a null correlation with C18:0. Moderate genetic correlations were observed between PUFA and the other FA traits, ranging from 0.19 ± 0.09 (with C18:0) to 0.59 ± 0.12 (with TFA). Moderate genetic correlations between PUFA and the other FA traits were observed also by Bastin et al. [6]. Strong genetic and phenotypic associations were estimated also between SCFA, MCFA, and the individual FA with the shortest chain length (i.e., C14:0 and C16:0), whereas LCFA resulted in a strong genetic and phenotypic correlation with C18:0 and C18:1. Our results are in accordance with estimates reported by Bastin et al. [6] and Petrini et al. [30]. Mostly weak or null genetic correlations were reported between TFA and the other FA traits; the large standard errors are due to the smallest data size.

### 3.3. Genetic and Phenotypic Correlations between FA and Milk Yield and Composition

Milk yield was negatively correlated, both at genetic and phenotypic level, with almost all FA traits, with the exception of a null genetic correlation with TFA (Table 3). Similar results were observed by Bastin et al. [6], who reported that a negative correlation was expected because of the effect of dilution. Thus, an increase of milk production is associated with a reduction of FA content. Moderate negative genetic correlations were obtained also by other authors [18,19,30]. Our findings indicate that selection for increased milk yield would affect almost all FA contents in the same way, as most of the genetic correlations with FA traits approached −0.50. Selection to increase fat percentage would increment all underlying FA in milk, with the exception of TFA. In particular, fat percentage showed the highest positive genetic correlation (>0.90) with SFA, SCFA, MCFA, and, accordingly, C14:0 and C16:0. Such high correlations were expected as these FA are the principal FA in fat. The strongest association between fat and SFA indicates that selection for increasing total fat content in milk would lead to an increased saturation level, which is undesirable from the nutritional point of view. Our results are in agreement with findings of Bastin et al. [6], who reported the highest correlations of fat percentage with SFA (0.97), SCFA (0.87), MCFA (0.95), and, among the individual FA, C14:0 (0.89) and C16:0 (0.92). Also, Penasa et al. [18] estimated the highest genetic correlation between milk fat and SFA (0.991), followed by milk fat and MUFA (0.812) and milk fat and PUFA (0.473). Similar pattern was observed by Petrini et al. [30], with fat percentage exhibiting the highest correlations with SFA (0.99) and C16:0 (0.98). As expected, our results differed to some extent to those of Mele et al. [25], due to the different expression of FA content. In that study [25] milk fat was negatively associated with C14:0 (−0.40 ± 0.18) and positively with C16:0 (0.74 ± 0.317) and C18:0 (0.28 ± 0.15). Protein and casein percentages were positively associated with all FA traits; in particular, the highest correlations (>0.70) were observed with SFA, MUFA, MCFA, and, among individual FA, C14:0 and C18:1. Also, Bastin et al. [6] obtained positive genetic correlations between protein percentage and FA, ranging from 0.18 (with C18:0) to 0.60 (with PUFA). Penasa et al. [18] reported positive associations between milk protein and FA groups, ranging from 0.441 (milk protein and PUFA) to 0.634 (milk protein and UFA). Genetic correlations between 0.33 (protein percentage and C18:0) and 0.69 (protein percentage and PUFA) were estimated by Petrini et al. [30]. Mele et al. [25] reported a weak association between milk protein and C14:0 (−0.12 ± 0.22), a negative association with C18:0 (−0.41 ± 0.19), and a positive correlation with C16:0 (0.60 ± 0.25). Lactose showed null to weak negative genetic and phenotypic correlations with FA. Petrini et al. [30] reported weak to moderate positive genetic correlations between lactose percentage and all FA traits analyzed. In particular, lactose was weakly correlated with SFA, C16:0, C18:0, and C18:1 (<0.15), and moderately correlated with UFA, MUFA, and PUFA (around 0.25). Urea had mostly moderate positive genetic and phenotypic correlations with almost all FA, with the exception of a negative correlation with TFA.

### 3.4. Genetic and Phenotypic Correlations between Fatty Acids and Milk Somatic Cells

Somatic cell score showed only a weak negative genetic correlation with PUFA and SCFA, whereas DSCC was not correlated with FA (Table 4). These findings suggest that genetic selection to improve resistance to mastitis, and possible inclusion of DSCC in selection index, would not affect FA content in milk. Bastin et al. [31] reported low genetic correlations between SCS and FA traits, ranging from −0.11 to 0.03. Although low, such correlations were not null as standard errors of estimates were ≤0.01. Overall, weak correlations of SCS with FA were observed also by Lopez-Villalobos et al. [9], except for moderate negative associations with C4:0 (−0.25), C6:0 (−0.21) and SCFA (−0.26). Genetic correlations between SCS and FA were estimated also by Petrini et al. [30], who reported weak negative correlations with SFA, C16:0 and C18:0, and weak positive correlations with UFA, MUFA, PUFA, and C18:1. Although such correlations were low and standard errors high, results are in line with findings of Randolph and Erwin [32], who observed higher concentrations of free FA and SCFA (4 to 12 carbons) and lower concentrations of LCFA (16 and 18 carbons) in mastitic than in normal milk. Changes in FA concentrations can be explained by variation in the permeability of the mammary gland tissue or modification of the synthesis of lipids. Weak to moderate positive daily genetic correlations between SCS and FA groups were estimated by Fleming et al. [20], possibly as a result of a moderate positive genetic correlation between SCS and fat percentage (0.341).

### 3.5. Genetic Selection on Milk FA Profile

In the last years, the nutritional quality of milk and dairy products has aroused considerable interest in the consumers. For this reason, it is of valuable importance to explore the feasibility of including FA in breeding programs, with the aim of modifying the proportions of FA in milk and improve the nutritional and healthy quality of fat. To be included in a selection program a trait must meet several criteria. In particular, it should be easily measurable at low cost and in a routine way, sufficiently variable and heritable, and economically important [33]. Milk FA content seems to fulfill these criteria. Thanks to mid-infrared spectroscopy, which has been widely used to determine milk composition traits (e.g., fat, protein, casein, lactose, and urea), and the development and implementation of FA calibration equations [34], it is now possible to routinely measure the detailed FA content in the frame of monthly recording system. In accordance with the literature [6,7,19], our findings indicate that FA present sufficient genetic variability and are moderately heritable (Table 1); thus, they can be exploited for breeding purposes. Genetic selection to improve the quality of fat (e.g., decrease SFA and increase Omega-3 and PUFA) will lead to milk with favorable FA profile for human health [1]. In addition, milk FA composition strongly influences the nutritional and organoleptic properties of milk, affecting in turn the technological characteristics of the milk itself and its derivatives [4]. Given the strong genetic correlations among some FA groups or individual FA (Table 2), mostly due to the similar origin, a careful evaluation should be performed to decide which FA should be included in a potential selection index. In addition, the association with other milk traits should be taken into account. In our study, a negative genetic correlation was observed between FA and milk yield, whereas moderate to strong positive correlations were reported with fat, protein and casein percentages (Table 3). Thus, the actual selection on milk production and composition in Italian Holstein cattle is affecting also FA composition. Nevertheless, to improve the nutritional quality of milk, selection should be performed on specific FA (e.g., Omega-3 and PUFA) and not on total fat content, which showed the strongest association with SFA. Genetic and genomic selection on FA profile is therefore feasible [35,36]; however, a clear breeding objective should be established. In addition, a new milk payment system based on the concentration of some FA should be considered to persuade farmers to improve milk fat profile [5].

## 4. Conclusions

In the present study we explored the genetic correlations among FA, and between FA profile and several milk traits. Genetic correlations among FA were moderate to high, according to their common origin (i.e., from the blood or synthesized de novo by the mammary gland) or their grouping according to saturation or chain length. Moreover, correlations among FA groups were in line with correlations among individual FA. Moderate negative genetic correlations were estimated between FA and milk production, whereas moderate to strong positive correlations were reported with fat, protein and casein percentages, indicating that the current breeding program in Italian Holstein cattle is affecting also all FA groups, not only the desired ones. No association with DSCC and low association with SCS suggested that selection on FA profile would not affect selection on resistance to mastitis and vice versa. Nevertheless, a certain caution should be taken in the interpretation of these results, which rely on test-day data, as other factors (e.g., diet, type of farming system) might affect the FA profile.

## Figures and Tables

**Table 1:** Descriptive statistics, additive genetic variance (σ^2^_a_), cow permanent environmental variance (σ^2^_pe_), residual variance (σ^2^_e_), estimated heritability (h^2^, with SE: standard error), and coefficient of additive genetic variation (CV_a_) of fatty acid (FA) profile, milk yield and composition, and milk somatic cells.

Trait	N	Mean (SD)	σ^2^_a_	σ^2^_pe_	σ^2^_e_	h^2^ (SE)	CV_a_ (%)
Groups of FA (g/100 g milk)							
SFA	35,206	2.62 (0.59)	0.05277	0.04842	0.16735	0.20 (0.02)	8.8
MUFA	35,052	0.98 (0.24)	0.00280	0.00358	0.03454	0.07 (0.01)	5.4
PUFA	35,238	0.10 (0.03)	0.00003	0.00005	0.00038	0.07 (0.01)	5.9
SCFA	35,219	0.41 (0.12)	0.00175	0.00181	0.00636	0.18 (0.02)	10.1
MCFA	35,221	1.74 (0.40)	0.02645	0.02309	0.06453	0.23 (0.01)	9.3
LCFA	35,084	1.33 (0.36)	0.00605	0.00878	0.07435	0.07 (0.01)	5.8
TFA	15,832	0.05 (0.04)	0.00002	0.00002	0.00079	0.02 (0.01)	8.2
Individual FA (g/100 g milk)							
C14:0 (myristic)	34,937	0.46 (0.10)	0.00143	0.00138	0.00405	0.21 (0.02)	8.2
C16:0 (palmitic)	34,909	1.16 (0.27)	0.01156	0.00942	0.03344	0.21 (0.02)	9.3
C18:0 (stearic)	34,805	0.39 (0.11)	0.00067	0.00121	0.00675	0.08 (0.01)	6.6
C18:1 (oleic)	34,762	0.88 (0.25)	0.00269	0.00349	0.03434	0.07 (0.01)	5.9
Milk yield and composition							
Milk yield (kg/day)	35,315	32.4 (10.9)	3.18197	16.89290	29.90190	0.06 (0.01)	5.5
Fat (%)	34,689	3.92 (0.78)	0.08054	0.08169	0.31547	0.17 (0.02)	7.2
Protein (%)	35,240	3.43 (0.42)	0.02505	0.02104	0.04796	0.27 (0.02)	4.6
Casein (%)	35,258	2.68 (0.34)	0.01773	0.01523	0.03120	0.28 (0.02)	5.0
Lactose (%)	35,311	4.76 (0.24)	0.00928	0.01052	0.02366	0.21 (0.02)	2.0
Urea (mg/100 mL)	35,270	23.7 (5.8)	1.94079	1.87956	10.35570	0.14 (0.01)	5.9
Milk somatic cells							
SCS (units)	34,795	3.17 (1.90)	0.15763	1.07864	1.76470	0.05 (0.01)	12.5
DSCC (%)	30,208	67.0 (16.7)	19.45550	57.72760	144.50400	0.09 (0.01)	6.6

N = number of records; SD = standard deviation; SFA = saturated fatty acids; MUFA = monounsaturated fatty acids; PUFA = polyunsaturated fatty acids; SCFA = short-chain fatty acids; MCFA = medium-chain fatty acids; LCFA = long-chain fatty acids; TFA = *trans* fatty acids; SCS = somatic cell score; DSCC = differential somatic cell count.

**Table 2:** Genetic (above diagonal, with standard error) and phenotypic (below diagonal) correlations between fatty acids (FA).

Trait	SFA	MUFA	PUFA	SCFA	MCFA	LCFA	TFA	C14:0	C16:0	C18:0	C18:1
Groups of FA											
SFA	-	0.67 (0.03)	0.31 (0.07)	0.93 (0.01)	0.98 (0.00)	0.73 (0.03)	−0.02 (0.14)	0.96 (0.01)	0.97 (0.00)	0.84 (0.03)	0.64 (0.04)
MUFA	0.68	-	0.58 (0.06)	0.59 (0.05)	0.64 (0.05)	0.91 (0.02)	−0.07 (0.18)	0.66 (0.05)	0.63 (0.05)	0.67 (0.05)	0.98 (0.00)
PUFA	0.51	0.74	-	0.44 (0.06)	0.23 (0.07)	0.38 (0.08)	0.59 (0.12)	0.41 (0.06)	0.21 (0.07)	0.19 (0.09)	0.55 (0.07)
SCFA	0.90	0.55	0.55	-	0.87 (0.01)	0.67 (0.05)	0.11 (0.17)	0.89 (0.01)	0.85 (0.02)	0.76 (0.04)	0.58 (0.05)
MCFA	0.96	0.56	0.36	0.81	-	0.69 (0.04)	−0.15 (0.18)	0.96 (0.00)	0.98 (0.00)	0.78 (0.03)	0.61 (0.04)
LCFA	0.71	0.97	0.72	0.57	0.58	-	−0.22 (0.19)	0.66 (0.05)	0.67 (0.04)	0.80 (0.03)	0.94 (0.01)
TFA	0.20	0.30	0.45	0.29	0.07	0.25	-	0.02 (0.16)	−0.15 (0.12)	−0.03 (0.18)	−0.23 (0.18)
Individual FA											
C14:0	0.91	0.48	0.35	0.79	0.95	0.51	−0.02	-	0.94 (0.01)	0.69 (0.04)	0.60 (0.05)
C16:0	0.96	0.60	0.37	0.78	0.97	0.62	0.07	0.92	-	0.79 (0.03)	0.58 (0.05)
C18:0	0.75	0.86	0.67	0.61	0.65	0.91	0.25	0.54	0.69	-	0.67 (0.05)
C18:1	0.66	0.98	0.74	0.58	0.51	0.95	0.37	0.41	0.55	0.83	-

SFA = saturated fatty acids; MUFA = monounsaturated fatty acids; PUFA = polyunsaturated fatty acids; SCFA = short-chain fatty acids; MCFA = medium-chain fatty acids; LCFA = long-chain fatty acids; TFA = *trans* fatty acids.

**Table 3:** Genetic (with standard error) and phenotypic correlations of fatty acids (FA) with milk yield and composition.

Trait	Milk Yield		Fat		Protein		Casein		Lactose		Urea	
	Genetic	Phenotypic	Genetic	Phenotypic	Genetic	Phenotypic	Genetic	Phenotypic	Genetic	Phenotypic	Genetic	Phenotypic
Groups of FA												
SFA	−0.52 (0.07)	−0.22	0.98 (0.00)	0.97	0.69 (0.03)	0.42	0.70 (0.03)	0.45	−0.06 (0.06)	−0.14	0.29 (0.05)	0.23
MUFA	−0.50 (0.05)	−0.22	0.78 (0.03)	0.82	0.76 (0.05)	0.23	0.75 (0.05)	0.25	−0.05 (0.05)	−0.13	0.34 (0.08)	0.16
PUFA	−0.26 (0.10)	−0.14	0.40 (0.06)	0.62	0.62 (0.05)	0.18	0.63 (0.05)	0.21	0.02 (0.07)	−0.10	0.31 (0.08)	0.23
SCFA	−0.48 (0.07)	−0.11	0.92 (0.01)	0.85	0.65 (0.04)	0.37	0.66 (0.03)	0.41	−0.04 (0.06)	−0.05	0.34 (0.04)	0.23
MCFA	−0.54 (0.07)	−0.23	0.95 (0.01)	0.9	0.70 (0.03)	0.48	0.70 (0.03)	0.5	−0.08 (0.05)	−0.17	0.29 (0.05)	0.21
LCFA	−0.50 (0.03)	−0.20	0.83 (0.03)	0.84	0.69 (0.06)	0.19	0.68 (0.06)	0.22	−0.05 (0.05)	−0.09	0.41 (0.08)	0.2
TFA	0.06 (0.21)	−0.08	0.00 (0.19)	0.23	0.18 (0.14)	0.01	0.22 (0.16)	0.03	−0.11 (0.11)	−0.06	−0.38 (0.16)	−0.05
Individual FA												
C14:0	−0.51 (0.07)	−0.17	0.94 (0.01)	0.83	0.75 (0.03)	0.48	0.76 (0.03)	0.51	0.02 (0.05)	−0.06	0.33 (0.06)	0.21
C16:0	−0.51 (0.07)	−0.23	0.94 (0.01)	0.9	0.63 (0.03)	0.37	0.63 (0.03)	0.39	−0.03 (0.05)	−0.15	0.29 (0.06)	0.23
C18:0	−0.52 (0.03)	−0.22	0.87 (0.02)	0.84	0.53 (0.06)	0.16	0.53 (0.05)	0.19	−0.06 (0.07)	−0.11	0.25 (0.08)	0.16
C18:1	−0.48 (0.09)	−0.21	0.76 (0.03)	0.79	0.73 (0.05)	0.23	0.72 (0.05)	0.25	−0.11 (0.06)	−0.14	0.38 (0.08)	0.19

SFA = saturated fatty acids; MUFA = monounsaturated fatty acids; PUFA = polyunsaturated fatty acids; SCFA = short-chain fatty acids; MCFA = medium-chain fatty acids; LCFA = long-chain fatty acids; TFA = *trans* fatty acids.

**Table 4:** Genetic (with standard error) and phenotypic correlations between fatty acids (FA) and milk somatic cells.

Trait	Somatic Cell Score		Differential Somatic Cell Count	
	Genetic	Phenotypic	Genetic	Phenotypic
Groups of FA				
SFA	−0.06 (0.09)	0.06	0.04 (0.07)	−0.01
MUFA	−0.05 (0.12)	0.10	0.01 (0.10)	0.00
PUFA	−0.14 (0.11)	0.08	0.02 (0.10)	0.00
SCFA	−0.17 (0.09)	−0.02	−0.03 (0.08)	−0.03
MCFA	−0.05 (0.09)	0.07	0.01 (0.07)	−0.01
LCFA	−0.12 (0.12)	0.06	−0.02 (0.10)	−0.01
TFA	−0.08 (0.20)	0.05	0.08 (0.14)	0.01
Individual FA				
C14:0	−0.09 (0.09)	0.02	0.05 (0.08)	−0.02
C16:0	−0.03 (0.09)	0.10	0.04 (0.07)	0.01
C18:0	−0.03 (0.11)	0.08	0.01 (0.09)	−0.01
C18:1	−0.09 (0.12)	0.09	−0.04 (0.10)	0.00

SFA = saturated fatty acids; MUFA = monounsaturated fatty acids; PUFA = polyunsaturated fatty acids; SCFA = short-chain fatty acids; MCFA = medium-chain fatty acids; LCFA = long-chain fatty acids; TFA = *trans* fatty acids.
